# Exploration of the triceps surae muscle in ambulatory children with cerebral palsy using instrumented measurements of stiffness and diffusion tensor magnetic resonance imaging for muscle architecture

**DOI:** 10.1186/s12891-024-07890-4

**Published:** 2024-10-11

**Authors:** Alexandra Åhblom, Eva Pontén, Antea Destro, Sven Petersson, Ferdinand von Walden, Ruoli Wang, Cecilia Lidbeck

**Affiliations:** 1 Division of Paediatric Neurology, Department of Women’s and Children’s Health, Karolinska Institutet, Stockholm, Sweden; 2 Theme/Functional Area Occupational Therapy & Physiotherapy, Women’s Health and Allied Health Professionals, Karolinska University Hospital, Stockholm, Sweden; 3 Department of Paediatric Orthopaedic Surgery and Pediatric Neurology, Astrid Lindgren Children’s Hospital, Karolinska University Hospital, Stockholm, Sweden; 4 Department of Engineering Mechanics, KTH Moveability, Royal Institute of Technology, Stockholm, Sweden; 5 Department of Clinical Neuroscience, Karolinska Institutet, Stockholm, Sweden; 6 Department of Medical Radiation Physics and Nuclear Medicine, Karolinska University Hospital, Stockholm, Sweden

**Keywords:** Cerebral palsy, Children, Contracture, Fascicle length, Muscle architecture, Muscle volume, Observational study, Stiffness, Pennation angle

## Abstract

**Background:**

Musculoskeletal alterations causing reduced range of motion of the ankle joint are common in children with cerebral palsy (CP). Objective measurements of passive joint resistance and three-dimensional skeletal muscle volume and muscle architecture can lead to a comprehensive understanding of which factors influence joint range of motion.

**Research question:**

To investigate the relation between the passive dorsiflexion of the ankle joint, biomechanical contributing factors to the passive joint resistance, and muscular architectural properties of the triceps surae muscle in children with CP.

**Methods:**

In this cross-sectional observational study, 14 children with spastic CP (bilateral: 5, unilateral: 9, Gross Motor Function Classification System (GMFCS) level I:11, II:3) naïve to intramuscular tone reducing treatment, and 14 TD children were included. The passive dorsiflexion of the ankle was measured with a goniometer. Passive joint resistance and related parameters were estimated based on a biomechanical model and measurements using a motorized device, the Neuroflexor. Three-dimensional muscle architecture was quantified with diffusion tensor magnetic resonance imaging (DT-MRI).

**Results:**

In the CP group, the median [min, max] passive dorsiflexion was decreased in the most affected leg (MAL) compared to the less affected leg (LAL) (2.5° [-25°, 20°] vs. 12.5° [5°, 30°], *p* = 0.001). The stiffness coefficient (Nm/rad) in the MAL was significantly higher in children with CP compared to TD children (7.10 [3.39, 62.00] vs. 2.82 [1.24, 10.46], *p* = 0.015). Muscle architecture properties did not differ between CP and TD, except for pennation angle in the medial gastrocnemius (MG) of the MAL (CP 17.64° (2.29) vs. TD 21.46° (3.20), *p* = 0.017). The stiffness coefficient, in the MAL, correlated negatively to passive dorsiflexion (r_s_=-0.638) and pennation angle in medial gastrocnemius (r_s_=-0.964), and the non-linear coefficient (Non-linear 1) correlated negatively to the fascicle length of the medial gastrocnemius (r_s_=-0.857).

**Conclusion:**

This study shows that stiffness of the plantarflexors is related to decreased passive dorsiflexion of the ankle and muscle structure of the MG in high-functioning children with spastic CP. Assessments of how dynamic components as well as microscopic muscle alterations contribute to joint stiffness in the plantarflexors in individuals with CP are warranted.

**Trial registration:**

Retrospectively registered in ClinicalTrials.gov, NCT05447299. Observational study. Study start: 2019-01-15, register date: 2022-07-01.

**Supplementary Information:**

The online version contains supplementary material available at 10.1186/s12891-024-07890-4.

## Background

Cerebral palsy (CP) is the most prevalent childhood onset motor disorder, caused by disturbances in the developing brain, and leading to activity limitations [1]. Although the brain lesion is not progressive, the development of secondary musculoskeletal problems during growth is common [2, 3]. The ankle joint is affected by a decreased passive range of motion [4, 5], which contributes to limited mobility and propulsion also in high motor functioning children with CP [6].

Increased passive joint stiffness and restricted dorsiflexion have been reported to be related to the reduced lengthening ability of fascicles in the medial gastrocnemius, as captured by ultrasound [6]. However, it remains to be explored how comprehensive muscle architectural features of the plantarflexors contribute to the increased joint stiffness and restricted dorsiflexion. In addition to being affected by decreased range of motion, most children with CP experience spasticity of the plantarflexors [7]. Clinical assessments of spasticity typically rely on subjective scales, with questionable reliability and validity, such as the Modified Ashworth Scale [8, 9]. These manual tests involve lengthening of the muscle at a slow velocity for the full range of motion, and at a fast velocity to elicit spasticity [10, 11], but are not designed to discriminate between the various biomechanical factors (neural and non-neural) that contribute to increased joint resistance typically present in children with CP [12]. For this level of detailed information, a motorized assessment is recommended [13, 14]. The NeuroFlexor (NF) (Aggero MedTech AB, Älta, Stockholm, Sweden) is a motorized instrument that measures passive resistant force during passive isokinetic dorsiflexion at controlled slow and fast speeds [15]. Together with a biomechanical computational model, this motorized measurement enables a dissection of the contributors to the non-neural (muscle and joint) passive resistance torque during slow trials into stiffness, viscosity, and non-linear components.

In children with CP, the volume of the gastrocnemius muscle has been observed to be reduced as early as during infancy [16, 17] and there is some evidence suggesting that smaller muscle volumes could be related to restricted dorsiflexion [18]. The contribution of other morphological features of skeletal muscles to a restricted range of motion, such as alterations in muscle fibre length and pennation angle (PA) are so far inconclusive [18].

Conventional B-mode ultrasonography can be used to quantify muscle architecture parameters in 2D or 3D when combined with a motion tracking system. However, due to the inherent limitation of ultrasound in penetration depth, studies have predominantly been focused on the medial gastrocnemius muscle [19]. For the diffusion tensor magnetic resonance imaging (DT-MRI), the advantage is that it is a valid and reliable imaging technique with a large field of view and a high resolution. Moreover, it can quantify the 3D muscle architecture features of both superficial and non-superficial muscles [19, 20]. Combining a comprehensive architectural exploration with instrumented assessments of ankle joint resistance during slow passive stretch of the plantarflexors could provide valuable insights for deciphering increased passive joint resistance in children with CP.

The aim of the study was to investigate muscle volume and muscle architectural parameters of the plantarflexors at rest without stress or strain and to explore whether these parameters relate to the passive joint stiffness and passive dorsiflexion of the ankle. Our hypotheses were that the passive ankle joint resistance in CP is negatively correlated to passive ankle dorsiflexion, muscle volume, fascicle length, pennation angle, and is different from typically developing (TD) children. We also hypothesised that passive ankle dorsiflexion, joint resistance, and muscle architectural parameters were more severely affected in the most affected leg (MAL) in CP compared to the less affected leg (LAL) and compared to the TD children.

## Methods

### Data acquisition

The participants underwent assessments at two separate occasions at the Karolinska University Hospital, Stockholm Sweden prior to a planned botulinum toxin type A treatment. The first occasion included a physical examination and instrumented joint resistance assessment, and the second occasion involved a DT-MRI examination. In children with CP, the more affected leg (MAL) and the less affected leg (LAL) were determined by clinical assessments and the participants’ self-perception of side difference, which was consistent in all cases. For children with unilateral spastic CP (USCP), the MAL was first examined. For children with bilateral spastic CP (BSCP), the order of the assessment (MAL or LAL) was randomized. The typically developing (TD) children underwent measurements exclusively on their right leg.

### Participants

In this cross-sectional observational study 28 children participated: 14 with spastic CP (median age 8.6 years [range 5,8 to 13,4]), functioning at Gross Motor Function Classification System (GMFCS) level I: 11 II:3, and 14 TD children (median age 9,4 years, [range 6,2 to 14,8]. Participant characteristics are presented in Table 1. Children were consecutively recruited between the period of June 2018 and August 2022, when planned for a first-time injection with botulinum toxin type A, at the Paediatric Orthopaedic Department at Karolinska University Hospital in Stockholm, Sweden. The decision for treatment had been made by an orthopaedic surgeon based on increased plantarflexor tone that resulted in walking in equinus, and for some, also difficulty to wear orthotics. Inclusion criteria for the study were 1) a confirmed diagnosis of CP [20], 2) age between 6 and 15 years, 3) GMFCS-level I, II or III [21], 4) ability to voluntarily perform isolated plantarflexion and dorsiflexion of the foot, 5) unilateral - or bilateral spasticity in the plantarflexors according to clinical tests as described below [10], 6) no prior exposure to BoNT-A treatment, 7) ability to follow instructions to perform the tests. Exclusion criteria were (1) a total range of motion of the ankle less than 40°, (2) severe pain in the lower leg, (3) orthopaedic surgery or lower limb fracture within the past six months. All children who met the inclusion criteria accepted to participate in the study. A convenience sample of TD children, age 6–15 years, with no history of orthopaedic injuries were recruited as a reference group.

Parental written consent and the children’s verbal agreement were mandatory before participation. All procedures followed the standards of the Declaration of Helsinki. The study was approved by the Regional Ethical Review Board in Stockholm, (DNR: 2014-1829-31-4, with addendums 2016-286-32, 2018-2128-32, and 2022-0212-02) and is registered in ClinicalTrials.gov (NCT05447299).

For the NF measurements, the available dataset comprised data from 14 TD children and 12 children with CP. In the CP group two assessments from the MAL, and three from the LAL were excluded from the analysis due to technical errors during the NF measurement. NF measurements were performed bilaterally on 11 children with CP (BSCP: 3, USCP: 8) and unilaterally on one child (BSCP).

For the DT-MRI measurements, data were available from: Ten children with CP and eight TD children. In the CP-group, three were not eligible to undergo DT-MRI scans due to safety reasons, one child declined due to time constraints and data from one participant was excluded due to motion artefacts evident during the analysis. If a child was struggling to lay still, a decision was made to prioritize the more affected side to reduce scan time. This resulted in that DT-MRI scans were performed bilaterally on five children (BSCP: 1, USCP: 4) and unilaterally on five children (BSCP: 2, USCP: 3). In the TD-group, DT-MRI data for eight participants was included. The absence of data from the other six TD children is attributed to various reasons: two of these participants exceeded the set timeframe of three months in relation to the clinical examinations, one participant declined participation and data from three participants were excluded due to motion artefacts.

**Table 1:** Characteristics of participants typically developing (TD) and with cerebral palsy

	TD *n* = 14	CP *n* = 14			TD/CP MAL *p*-value
Age (years)	9.4 [6.2, 14.8]	8.6 [5.8, 13.4]			0.603
Weight (kg)	34.8 [21.6, 62.8]	29.8 [18.0, 44.3]			0.164
Height (cm)	139.5 [113.5, 171.0]	136.5 [113.0,152.0]			0.401
Boys/girls	6/8	8/6			0.706
BSCP/USCP	n/a	5/9			n/a
GMFCS level I/II	n/a	11/3			n/a
**Physical examination**	**TD**	**CP MAL**	**CP LAL**		**CP MAL/LAL** ***p*** **-value**
Passive ankle dorsiflexion° extended knee	n/a	2.5 [-25, 20]	12.5 [5, 30]		0.001*
Passive ankle dorsiflexion° flexed knee	n/a	12.5 [-10, 20]	20 [10, 30]		> 0.001*
MAS 0/1/+1/2/3/4	n/a	0/2/2/8/2/0	3/3/5/2/1/0 n/a		
SMC 0/1/2/3/4	n/a	0/2/4/4/2	0/0/1/5/6 n/a		

Median [min, max] of age, weight, height, and representation of sex, and spastic subtype bilateral- (BSCP) or unilateral spastic CP (USCP) and gross motor function classification system (GMFCS) in the group of children with cerebral palsy (CP). Passive ankle dorsiflexion with extended-, and flexed knee, spasticity with the Modified Ashworth scale (MAS), and selective motor control (SMC) according to Boyd and Graham, in the most affected leg (MAL), and the less affected leg (LAL). Non-parametric statistics was used (p-level 0.05). * Indicate significant difference. n/a = not applicable, °= degrees

### Physical examination

A physical examination was performed by two experienced paediatric physiotherapists, including measurements of height, weight, and length of the lower leg (proximal fibula to the end of the lateral malleolus) and the foot (posterior lateral malleolus to the distal fifth metatarsal). Maximum passive dorsiflexion of the ankle was assessed in individuals with CP using a goniometer, with measurements taken both with the knee flexed and extended. Spasticity in the plantarflexors was assessed according to the Modified Ashworth scale [10], and selective motor control during dorsiflexion of the ankle according to Boyd and Graham 1999 [22].

### Passive ankle joint resistance assessment

**Fig. 1:**
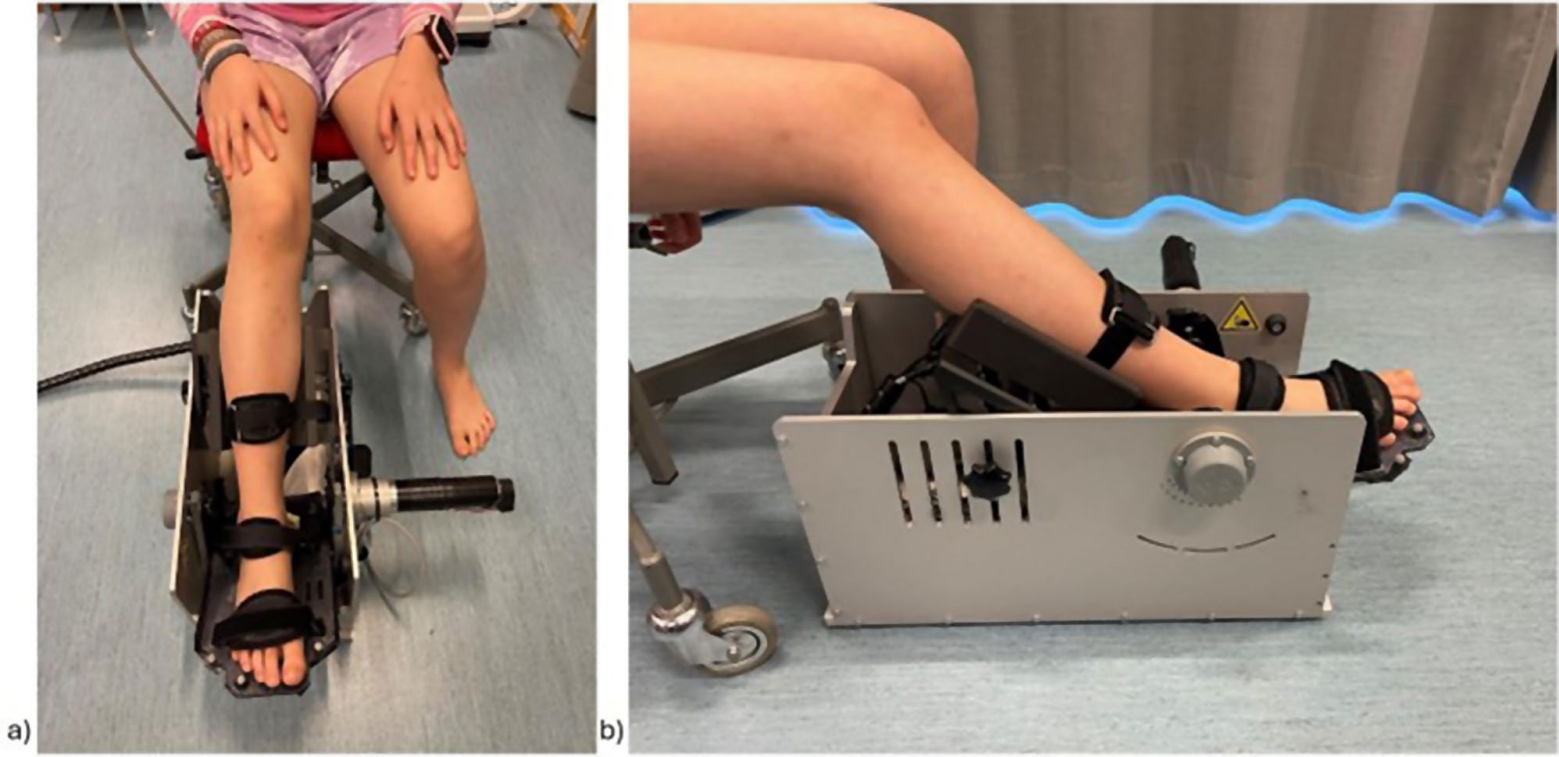
Illustration of test set-up for measurements of joint resistance with the NeuroFlexor. The foot rests on the foot plate with an adapted calf-support and is attached with Velcro-straps to ensure stability. Measurements are performed in a range from 35˚ plantarflexion to 5˚ dorsiflexion at a velocity of 5˚/sec, starting from a neutral position (0º) position. A force tranceducer records the total force (N) opposing the movement. **a**) Frontal view of the NeuroFlexor instrument **b**) Sagital view of the NF

Passive ankle joint resistance was instrumentally measured with the NeuroFlexor (NF) (Aggero MedTech AB, Älta, Stockholm, Sweden). The NF is a portable computer-controlled servo-motor instrument including a high-resolution step motor and motor controller, measuring the resistance force during passive isokinetic dorsiflexion at a predefined constant velocity [14, 23], which has been shown to give valid and reliable measures [15]. In this study, we performed NF assessment at a slow velocity of 5˚/s, which assumes to measure the ankle resistance only due to non-neural related contributors [15]. The foot plate and the calf support were customized to fit a paediatric population. During the assessment, participants were sitting on a chair, with 90° hip flexion and 30° knee flexion. One foot at a time was placed in the NF with the other foot placed on the floor and the child being as relaxed as possible (See Fig. 1 for set-up). The ankle range of motion during the measurement was typically set between 35° plantarflexion to 5° dorsiflexion. In two exceptions, due to impaired ability to dorsiflex above neutral, the NF was set to end with 5° plantarflexion, or with a neutral position (0°). Three out of 10 trials were chosen for further analysis according to the methods described by Lindberg et al. 2011 [23].

The passive ankle joint resistance torque ($\:{T}_{slow}$) was calculated by multiplying the measured resistance force from NF with a constant moment arm measured from the NF device. We assumed that the ankle resistant torque was purely generated by the passive viscoelastic elements of the muscle and connective tissues of the ankle [24]. A modified biomechanical model was therefore used consisting of an angle-dependent component i.e., the elasticity, and an angular velocity-dependent component i.e., viscosity. Four parameters: stiffness coefficient ($\:{k}_{p}$) and two non-linear coefficients ($\:{k}_{1}\:and\:{k}_{2}$), and viscosity coefficient ($\:{B}_{p}$) were then estimated using the following equation as Eq. (1) [25].

1 $$T_{slow}=k_{p}\cdot(\theta-\theta_{0})+B_{p}\cdot\dot{\theta}+k_{1}(exp(k_{2}\cdot(\theta-\theta_{0}))+T_{0}$$

where $\:\theta\:$, $\:\dot{\theta\:}$ and $\:{\theta\:}_{0}$ denote the angle, angular velocity, and initial angle, $\:{T}_{0}$ the measurement offset.

Non-linear least-squares optimization was used to approximate the four parameters [$\:{k}_{p}$, $\:{B}_{p}$, $\:{k}_{1}$, $\:{k}_{2}$] for three selected trials using Matlab^®^ R2020a and R2021a. Non-linear least-squares optimization is a form of least-squares optimization to fit an observation with a model with a set of unknown parameters. The initial values of these parameters were modified from the literature [14] as following:

$$\begin{aligned}Kp\hspace{0.17em}&=\hspace{0.17em}0.5\:Nm/rad,\:Bp =\hspace{0.17em}0.1\:Nms/rad,\cr&\:k1\hspace{0.17em}=\hspace{0.17em}0.5\:Nm,\:k2 =\hspace{0.17em}0.5\:rad\hspace{0.17em}^{-1\:}\end{aligned}$$

The initial 0.5s and the last 2s of the slow movement were eliminated from the analysis to avoid the acceleration and deceleration phases of the measurement. The fitness of the biomechanical model to the measured data is analysed by the Variance Accounted For (VAF) as Eq. (2) [26]:

2 $$\:VAF=\left(1-\:\frac{\varSigma\:{\epsilon\:}^{2\:}\left(t\right)}{\varSigma\:{{T}_{measure}}^{2}\left(t\right)}\right)\cdot\:100\%\:$$

^∞^ With $\:{\epsilon\:}\left(t\right)$ denoting the error, i.e., the difference between the measured resistance torque, $\:{T}_{measure}\left(t\right)$ and the modelled resistance torque $\:{T}_{slow}\left(t\right)$, over t = 1, …, N samples.

### 3D muscle volume and muscle architecture assessments of the calf muscle

**Fig. 2:**
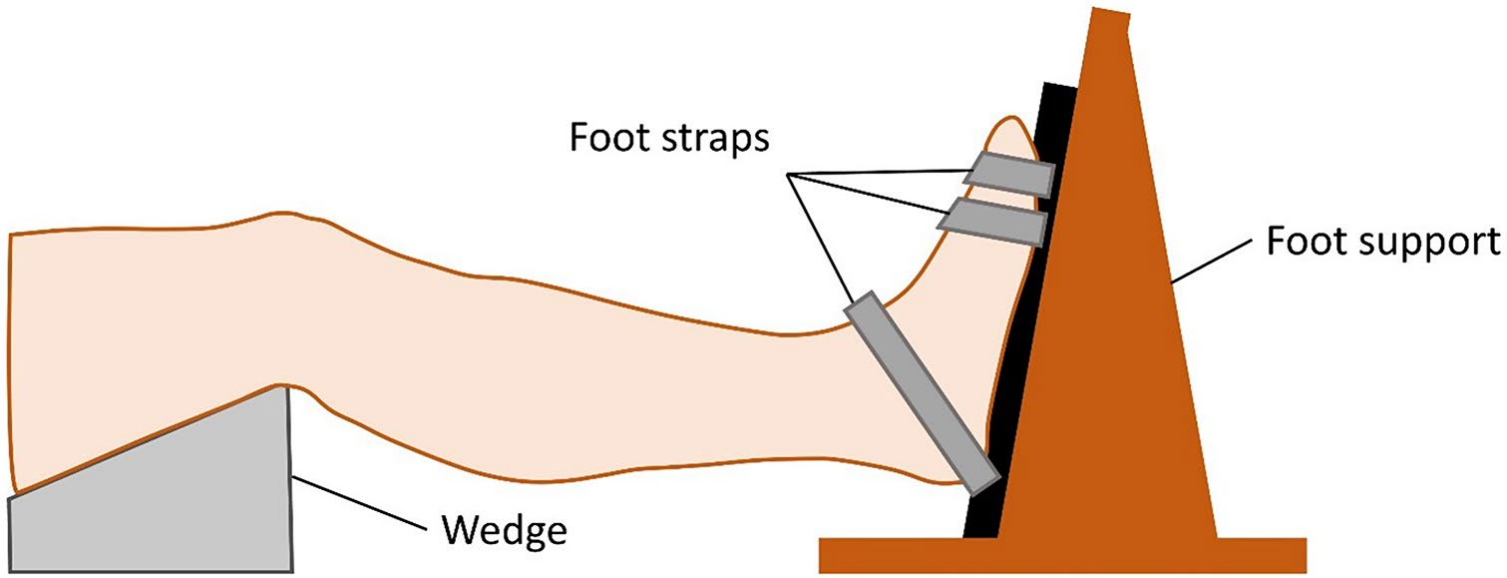
Illustration of the experimental set up for DT-MRI examination. Original figure by Wang et al. 2023 [27], https://creativecommons.org/licenses/by/4.0/

Plantarflexors were scanned using a 3 Tesla MRI scanner (Magnetom Prisma-fit, Siemens Healthineers, Erlangen, Germany) in a supine position with 20˚ knee flexion and 10˚ ankle plantar flexion. See Fig. 2 for set-up.

A T1-weighted anatomical scan was obtained with the following settings: TSE sequence, TR/TE 605/23 ms, transverse FOV 201 × 340 mm², slice thickness 5 mm, voxel size 0.7 × 0.7 × 5.0 mm^3^, flip angle 120˚, scan time 1:29 min. The DT-MRI was obtained with the following settings: EPI sequence, TR/TE 5800/63 ms, sagittal FOV 350 × 350 mm², slice thickness 2.5 mm, voxel size 2.5 × 2.5 × 2.5 mm^3^, 20 diffusion directions, EPI factor 140, b-value = 500 s/mm2 (reference image with b = 0 s/mm^2^), scan time 8:15 min.

**Fig. 3:**
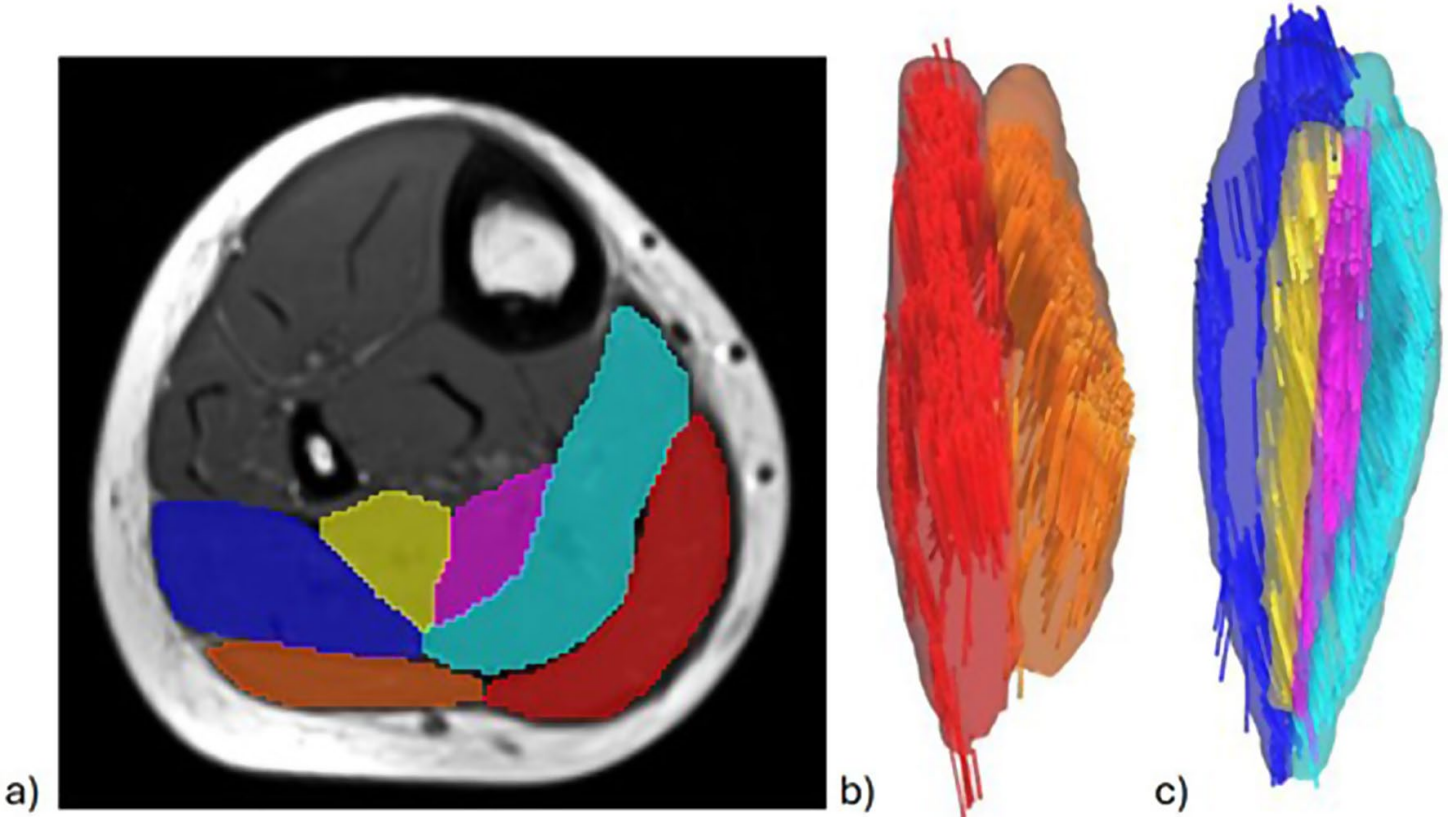
Diffusion tensor magnetic resonance imaging of the lower leg. Overview of methods to measure muscle architecture using diffusion tensor magnetic resonance imaging (DT-MRI) **a**) The transverse view of the anatomical DT-MRI slice approximately midway between the ankle and knee. Two compartments of gastrocnemius and four compartments of soleus were segmented consisting of medial gastrocnemius (MG, in red), lateral gastrocnemius (LG, in orange), medial posterior soleus (MPS, in turquoise), lateral-posterior soleus (LPS, in blue), lateral-anterior soleus (LAS, in yellow), medial-anterior soleus (MAS, in purple). **b**) 3D reconstructed fascicle tracks of MG and LG, anterior view, based on DT-MRI data **c**) 3D reconstructed fascicle tracks of MPS, LPS, LAS and MAS, anterior view, based on DT-MRI data

The post-processing of the T1 and DT-MRI was modified based on previous studies [27, 28]. Briefly, muscle boundaries were manually outlined on each slice of the T1-weighted images, and a 3D triangulated surface model of the muscle was generated [29]. Two compartments of the gastrocnemius, the lateral and the medial, were segmented. Four compartments of the soleus muscles were also segmented: medial anterior soleus, lateral anterior soleus, medial posterior soleus, and lateral posterior soleus (Fig. 3) [30]. The muscle volume for the triceps surae was calculated, including both the whole gastrocnemius and the soleus muscle.

The DT-MRI image underwent initial denoising using a Local Principal Component Analysis (LPCA) filter [31]. Subsequently, the filtered data were imported into DSI-Studio (http://www.dsi-studio.labsolver.org) for fibre tracking. During the tractography, 1000 fibre tracts were generated throughout the whole muscle volume per compartment. All tracts were then exported into MATLAB (version R2017b, The MathWorks Inc., Natick, MA, USA) and overlaid onto the 3D triangulated surface model. Each muscle fibre tract was fitted with a three-dimensional third-order polynomial curve, which included extensions at the endpoints by linear projections onto the muscle surface [32]. The polynomial curve, along with the extensions, is referred to as a fascicle. The reported fascicle length in mm was calculated as the mean length of 1000 fascicles in each muscle compartment and normalized to the participant’s body height (in mm) [33]. Superficial and deep pennation angle at each of the fascicle’s attachments to an aponeurosis was calculated as 90° minus the mean angle between a vector parallel to the endpoint’s slope and the normal vectors of all triangles of the surface model within a radius of 1.5 mm around the endpoint. The final pennation angle is the mean of the superficial and deep pennation angles (°) [32]. Pennation angles and fascicle lengths were calculated for all tracts and are presented as an averaged value within the whole volume. Muscle volume is presented as absolute millilitres (mL), and as normalized to height and weight (mL/ (kg * cm)). The physiological cross-sectional area (PCSA) was calculated as: PCSA = cos (pennation angle) * (muscle volume/fascicle length) and is presented in the unit of cm^2^ [34].

### Statistical analysis

All statistical analyses were performed using IBM corp. SPSS Statistics version 27.0 (Armonk, NY, USA). The sample size determination was based on pilot data from 10 TD children and prior literature, considering an effect size of 52 N for passive resistance at ankle joint, a power of 0.8, and an alpha level of 0.05. It is noteworthy to mention that there was no previous study using NF to measure ankle joint resistance or directly reporting ankle resistance during a slow and constant velocity stretching in children with CP. We estimated the effect size as 1.5 times of the mean joint resistance in the TD group (34 N). This analysis determined that a minimum of eight participants in each group was required [35]. The significance level was set to 0.05. Descriptive data on explanatory variables is presented as mean (SD) for normally distributed data, or median [min, max] for non-normally distributed data.

Firstly, a Kolmogorov-Smirnov test was conducted to assess the normal distribution of the data. For non-normally distributed data, Mann-Whitney U test was used to examine potential differences between groups, and a paired Wilcoxon test to assess within-group differences, with respect to MAL and LAL in the CP group. For normally distributed data, a Welch t-test was performed for comparisons between the groups, and a paired t-test was used for within group analysis. Spearman’s rho correlation coefficient (r_s_) was used to explore potential correlations between passive ankle joint resistance parameters ($\:{k}_{p}$, $\:{k}_{1}\:and\:{k}_{2}$), spasticity and maximum passive dorsiflexion, muscle volume, and muscle architecture parameters (pennation angle and fascicle length of gastrocnemius and soleus), of the MAL in the CP group. The following interpretation was used for the size of the correlation: a range from 0.00 to 0.30 indicating poor correlation, 0.30–0.50 fair, 0.50–0.70 moderate, 0.70–0.90 very strong, and a value of 1 signifying perfect correlation, and considered significant with p/alpha-value < 0.05.

## Results

### Physical examination

Data from the physical examination are presented in Table 1. In the CP group the passive ankle dorsiflexion was significantly less in the MAL compared to LAL for both extended (2.5˚ [-25 ˚, 20 ˚] vs. 12.5˚ [5 ˚, 30 ˚], *p* = 0.001) and flexed knee positions (12.5˚ [-10 ˚, 20 ˚] vs. 20˚ [10˚, 30˚], *p* = 0.001).

#### Passive ankle joint resistance measured with the NeuroFlexor

**Fig. 4:**
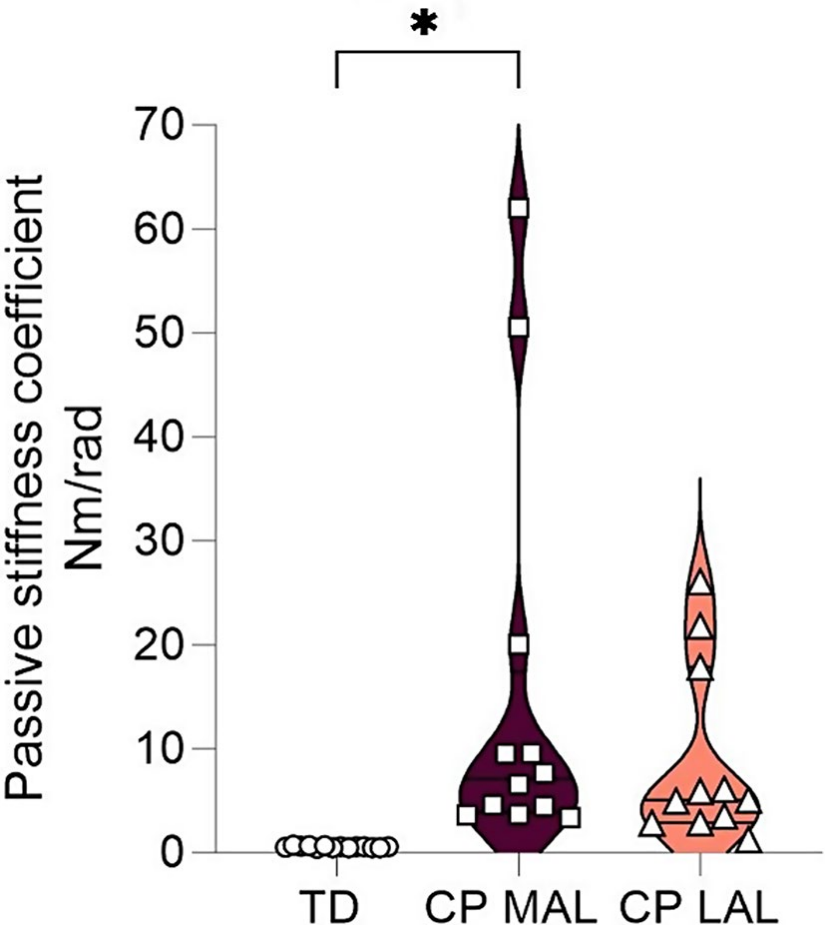
Passive stiffness coefficient in the lower leg. Comparison of stiffness coefficient (Nm/rad), median [min, max), based on NeuroFlexor lower leg assessment in typically developing children (TD) (*n* = 14), and in children with cerebral palsy (CP); in the most affected leg (MAL) (*n* = 12), and the less affected leg (LAL) (*n* = 11). * Indicate significant difference of *p* < 0.05

The modelled torque had a good fit to the measured total resistance ankle torque (TD-group; VAF: 0.996, CP MAL VAF: 0.998, CP-group; LAL VAF: 0.991). The stiffness coefficient was significantly higher in the CP-group in MAL compared to the TD-group (7,10 [3.39, 62.00] vs. 2,82 [1.24, 10.46], *p* = 0.015), but no significant differences were found between LAL and TD (*p* = 0.149), or between MAL and LAL in the CP-group (7,10 [3.39, 62.00] vs. 5.05 [1.25, 26.05], *p* = 0.075) (Fig. 4). The coefficients $\:{B}_{p}$, $\:{k}_{1}$, and $\:{k}_{2}$ did not differ significantly between the CP-group and the TD-group (*p* = 0.374, *p* = 0.168, and *p* = 0.538), or with respect to MAL and LAL in the CP-group (*p* = 0.386, *p* = 0.386, and *p* = 0.333 respectively). Data is presented in Table 2.

**Table 2:** Passive ankle joint resistance parameters

	CP MAL	*n*	CP LAL	*n*	TD	*n*	TD/CP MAL *p*-value	TD/CP LAL *p*-value	CP MAL/ LAL *p*-value
Stiffness coefficient K_p_ (Nm/Rad)	7.10 [3.39, 62.00]	12	5.05 [1.25, 26.05]	11	2.82 [1.24, 10.46]	14	0,015*	0.149	0.075
Viscosity B_p_ (Nm • s/Rad)	0.13 [0.10, 0.97]	12	0.11 [0.10, 0.34]	10	0.12 [0.6, 0.28]	14	0.374	0.841	0.386
Non-linear 1 $\:{\varvec{k}}_{1}$ (Nm)	0.24 [0.01, 2.91]	12	0.09 [0.01, 16.97]	10	0.07 [0.00, 3.66]	13	0.168	0.410	0.386
Non-linear 2 $\:{\varvec{k}}_{2}$ (rad^− 1^)	7.18 [3.05, 9.75]	12	5.96 [2.62, 8.69]	10	6.33 [2.24, 9.74]	13	0.538	0.446	0.333

Median [min, max] of non-neural components: stiffness coefficient, viscosity, non-linear 1 and non-linear 2 of passive ankle joint resistance from measurements with the NeuroFlexor in children with cerebral palsy (CP) in the most affected leg (MAL) and the less affected leg (LAL), and in typically developing (TD) children. * Indicate significant difference (p-level < 0.05) analysed with nonparametric statistics

#### 3D muscle volume and muscle architecture parameters measured with DT-MRI

**Fig. 5:**
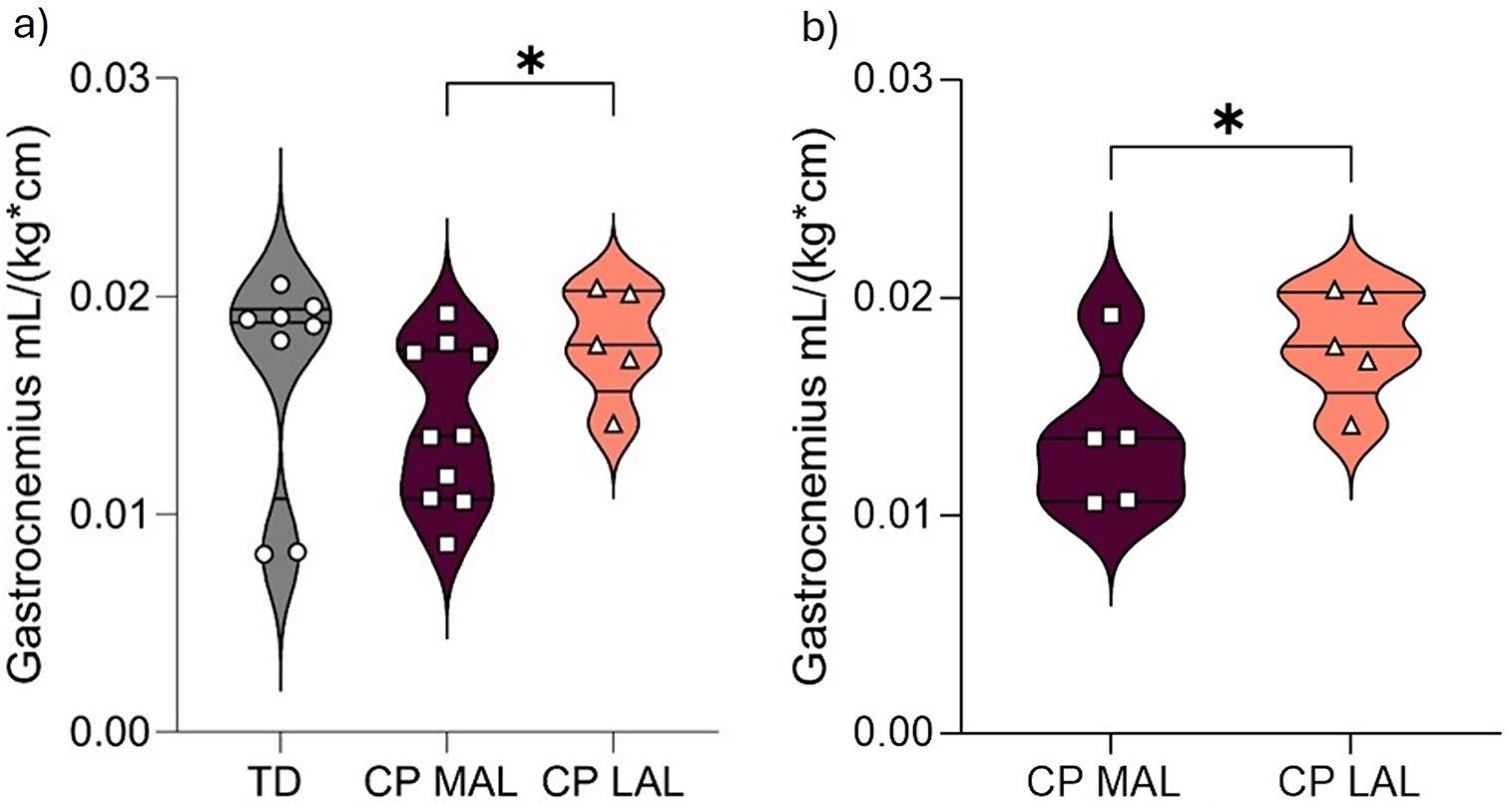
Muscle volume of total gastrocnemius. Normalized muscle volume (mL/kg*mm) of total gastrocnemius measured with diffusion tensor magnetic resonance imaging (DT-MRI), mean (sd) **a**) in typically developing children (TD) (*n* = 8) and in children with cerebral palsy (CP) in the most affected leg (MAL) (*n* = 10), and in the less affected leg (LAL) (*n* = 5), and **b**) in the children with CP with bilateral measurements (*n* = 5). * Indicate significant difference of *p* < 0.05

Complete data for the 3D muscle volume and muscle architecture parameters are presented in Supplementary Table 1 (S1). Absolute muscle volume did not significantly differ between the CP-group (MAL) and TD-group in either gastrocnemius (*p* = 0.806), soleus (*p* = 0.625) or total triceps surae (*p* = 0.700). In the CP-group, absolute muscle volume in gastrocnemius, soleus, and triceps surae were significantly smaller in MAL than in LAL (*p* = 0.013, *p* = 0.018, and *p* = 0.014). When normalized to height and weight (mL/(kg*cm)) muscle volume of the whole gastrocnemius was smaller in MAL compared to LAL (0.023 (0.006) vs. 0.03 (0.004), *p* = 0.041), (Fig. 5), but not for soleus or the triceps surae.

The normalised fascicle length of the medial and lateral gastrocnemius compartments did not significantly differ between the CP-group (MAL) and TD-group (*p* = 0.724 and *p* = 0.473 respectively). In the soleus, the fascicles were significantly longer in the lateral posterior compartment in the CP-group compared to the TD-group (0.016 (0.004) vs. 0.011 (0.003), *p* = 0.20). In the CP group, MAL and LAL fascicle lengths did not differ in any muscle.

The pennation angle in the medial gastrocnemius was significantly smaller in the CP-group compared to the TD-group (17.64 (2.29) vs. 21.46 (3.20), *p* = 0.017). No significant differences were observed in the lateral gastrocnemius or soleus muscles. In the CP-group, the pennation angle in the lateral posterior soleus was significantly smaller in MAL compared to LAL (22.77 (6.48) vs. 23.88 (7.92), *p* = 0.024). No other significant differences were observed in the remaining compartments of the soleus muscle.

The normalised PCSA did not differ in any compartment of gastrocnemius or soleus between the CP-group and TD-group. In the CP-group between MAL and LAL, there was a significant difference where PCSA for MAL was smaller in the medial gastrocnemius (24.88 [12.67, 33.64] vs. 34.58 [17.69, 35.88], *p* = 0.028). No other significant differences were observed.

#### Correlations

**Fig. 6:**
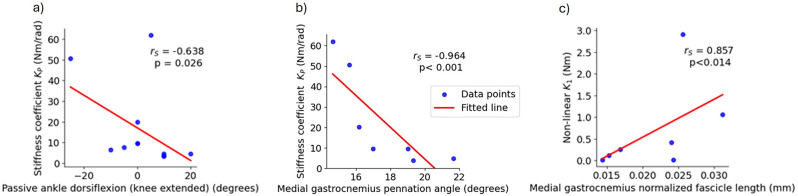
Correlations of ankle joint stiffness parameters and passive dorsiflexion, and medial gastrocnemius muscle architecture. Spearman’s rho (r_s_) correlations of the ankle passive joint stiffness coefficient $\:{(k}_{p})$ (Nm/rad) and **a**) passive maximal ankle dorsiflexion with extended knee (degrees) (*n* = 12) and **b**) medial gastrocnemius pennation angle (degrees) (*n* = 7), and of Non-linear 1 $\:{(k}_{1})$ and medial gastrocnemius fascicle length (*n* = 7) of the most affected leg in children with cerebral palsy

In CP MAL, the stiffness coefficient $\:{(k}_{p})$ had a moderate negative correlation to ankle passive dorsiflexion (r_s_=-0.638, *p* = 0.026), and a very strong negative correlation to the pennation angle in the medial gastrocnemius (r_s_=-0.964, *p* < 0.001). Non-linear 1 ($\:{k}_{1}$) had a very strong negative correlation to fascicle length in medial gastrocnemius (r_s_=-0.857, *p* < 0.014) (Fig. 6a, b and c). There were no correlations between the joint stiffness parameters and spasticity assessed with modified Ashworth scale or with muscle volume.

## Discussion

This study examined ankle joint stiffness in children with spastic cerebral palsy, using comprehensive and advanced methodologies including clinical examination, biomechanical measurements, muscle volume, and characterization of muscle architecture. The results reveal an increased passive stiffness coefficient in the plantarflexors of children with spastic CP compared to a group of TD children. The observed biomechanically stiffer plantarflexors in children with CP were related to decreased passive ankle dorsiflexion, to smaller pennation angle in the medial gastrocnemius and to longer fascicles in part of the soleus, but not to plantarflexor muscle volume. Fascicles with smaller pennation and thus oriented more in the line of pull, may explain some of the increased stiffness, but inherent microscopic changes of muscles in children with spastic CP may also contribute to the resistance when the foot is slowly moved in dorsiflexion.

There was a more than two-fold increase in the passive stiffness coefficient in the MAL of children with CP as compared to TD children. Our results are in line with published literature using less rigorous, manual methods for measurement of plantarflexor resistance [36, 37] but provide a greater level of detailed differentiation between each biomechanical contributor. When the NF measurement is performed, the passive resistance is assessed at the level of the joint, and a higher stiffness coefficient most likely indicate a higher muscle stiffness. Increased single fibre stiffness [38], and elevated skeletal muscle collagen content have been reported by several independent research groups, in both the upper and lower limbs [39–42]. Stiffness of fibre bundles with and without the surrounding extracellular matrix has been assessed in the lower limbs in the hamstrings, where the extracellular matrix was stiffer in the CP group than in the TD-group, and in the gastrocnemius and soleus, where the fibres themselves were stiffer in the CP-group than in the TD-group [40, 43]. Mathematical modelling of the muscle stiffness in CP has suggested that the volume fraction of extracellular matrix may influence muscle stiffness, but that sarcomere length and extracellular matrix stiffness itself may not be the largest contributor to increased muscle stiffness [44]. Contrary to our hypothesis there was no significant difference in stiffness between MAL and LAL in our group of children with CP.

We found a notable and significant discrepancy between MAL and LAL regarding passive ankle dorsiflexion in the CP-group. For the LAL the ankle passive dorsiflexion fell within the mean range reported in published reference values for children free from conditions that could limit joint mobility [43]. Previously, a negative association between stiffness around the ankle and the total range of motion, was described by de Gooijer-van de Groep and co-workers, utilizing a motorized instrumented measurement technique [45]. In line with their observation, we showed an association between the stiffness coefficient and decreased passive dorsiflexion of the ankle in MAL in our cohort of children with CP. Other studies on children with spastic CP using 2D ultrasound have shown that the total muscle tendon unit of the gastrosoleus complex is shorter compared to TD peers, with the tendon being longer and the muscle belly shorter. Movement of the gastrocnemius muscle-tendon junction during both passive and active ankle rotation have indicated that the gastrocnemius muscle lengthens less and is stiffer than the tendon in CP [46–48]. Based on our findings, we propose that, the increased stiffness observed relates to decreased passive dorsiflexion of the ankle. Previous research on the wrist joint suggested that an earlier exponential rise of the resistance force, caused by a higher $\:{k}_{1}$ may relate to the limited passive wrist extension [14]. However, no such correlation was observed in the ankle in this study. It is worth mentioning that during the NF measurements, the knee position was standardized to 30 degrees of flexion as it was our intention to perform measurements in the middle range of motion considering the comfort and safety of a motorized measurement. It was thereby unlikely that the ankle would be moved to its end range inducing an exponential rise of the resistance force. The joint position during measurements therefore might have contributed to the undifferentiated $\:{k}_{1}$ between the groups.

Associations were found between some muscle architecture parameters of the medial gastrocnemius and stiffness in our cohort of high functioning children with CP. A smaller pennation angle and longer fascicle length in the medial gastrocnemius thereby might contribute to a higher stiffness. Somewhat surprisingly, less pronounced differences regarding muscle volume were found in this cohort of children compared to previous research [18, 49]. Nevertheless, within the group of children with CP, the MAL exhibited reduced muscle volume compared to LAL but not compared to the TD-group. A study by Willerslev-Olsen et al. revealed that impaired muscle growth preceded increased stiffness [50]. Consequently, reduced muscle growth may influence the development of contractures and thereby decreased range of motion [18]. In a review on muscle architecture in CP, findings of muscle alterations were shown to be somewhat inconsistent [2], and there is yet to be elucidated how the compositional changes are linked to stiffness. Contrary to our hypothesis, we found no significant differences regarding joint stiffness, muscle volume or muscle architecture in LAL in the CP-group compared to the TD-group.

### Limitations and strengths

Our study has focused on a small cohort of high-functioning children with spastic CP, limiting the generalizability of our findings to the broader CP population. Only children functioning at GMFCS-level I and II fulfilled the inclusion criteria during the recruitment period. Moreover, the CP group comprised both BSCP and USCP children, with a majority experiencing unilateral symptoms, a distribution that contrasts with the prevalence typically observed within the diagnosis [51]. In the analysis, the stratification was based on MAL and LAL sides, but the LAL group encompasses both affected and minimally affected sides, given its inclusion of cases representing both USCP and BSCP [52].

Measures of passive stiffness may be influenced by the range of motion of which the assessment are carried out [53]. Forces at the end of ankle dorsiflexion may hamper accurate measurements of stiffness. The technical requirement for the NF demands a range of motion > 40 degrees and therefor all included children with CP exceeded this limit. All children included in the measurements were naïve to intramuscular tone reducing treatment and previous orthopaedic surgeries, representing a natural history of the development of muscles in young individuals with CP. Further exploration is needed to understand the functional and mechanical importance of muscular architectural changes, neural contribution, and the effect of treatment.

## Conclusions

This study showed that stiffness of the plantarflexors was related to decreased passive dorsiflexion of the ankle and muscle structure of the medial gastrocnemius in high-functioning children with spastic CP. Assessments of how dynamic components such as stiffness at fast velocity as well as microscopic muscle alterations contribute to joint stiffness in the plantarflexors in individuals with CP are warranted.

## Electronic supplementary material

Below is the link to the electronic supplementary material.

Supplementary Material 1

## Data Availability

The datasets generated and analysed during the current study are not publicly available due to risk of comprising individual privacy but are available from the corresponding author on resoanable request.

## Abbreviations

CP: Cerebral palsy
NF: NeuroFlexor
DT-MRI: Diffusion tensor magnetic resonance imaging
MAL: Most affected leg
LAL: Less affected leg
USCP: Unilateral spastic CP
BSCP: Bilateral spastic CP
GMFCS: Gross Motor Function Classification System
TD: Typically developing
