# Supplementary material for: Exploration of the triceps surae muscle in ambulatory children with cerebral palsy using instrumented measurements of stiffness and diffusion tensor magnetic resonance imaging for muscle architecture
Source: BMC Musculoskelet Disord. 2024 Oct 11;25:803. doi: 10.1186/s12891-024-07890-4 (PMC11468337; doi:10.1186/s12891-024-07890-4)
Supplement: Supplementary file 1 — Supplementary Material 1 [file 12891_2024_7890_MOESM1_ESM.pdf]

Supplementary Table 1. Muscle architecture of the calf muscles

Mean (sd) or median [min, max] muscle volume (MV) normalized to body ratio, mL/ (kg* cm), fascicle length (FL) normalized to body height in mm, pennation angle in degrees, and physiological cross-sectional area in cm² in medial gastrocnemius (MG), lateral gastrocnemius (LG) in the most affected leg (MAL) or less affected leg (LAL) in children with cerebral palsy (CP), and in the right leg for typically developing (TD) children. Statistics were analysed with independent samples Welch T-test, or as indicated with (†) non-parametric Mann-Whitney U test.

Muscle volume (MV) in mL and normalized mL/ (kg* cm)	**TD** n=8	**CP MAL** n=10	**CP LAL** n=5	**TD/CP MAL** p-value	**TD/CP LAL** p-value	**CP MAL/LAL** p-value
MG MV	70.61(18.96)	57.59(25.19)	85.10(33.87)	.229	.417	.02*
MG MV: mL/ (kg cm)*	0.016(0.005)	0.014(0.004)	0.018(0.003)	.300	.491	.043*
LG MV	27.30(7.82)	36.27 (17.95)	40.89(19.30)	.179	.196	.036*
LG MV: mL/ (kg cm)*	0.006(0.002)	0.009(0.003)	0.009(0.002)	.061	.066	.975
Gastrocnemius MV	97.91(25.48)	93.86(42.46)	126.00(52.38)	.806	.312	.013*
Gastrocnemius MV: mL/ (kg* cm)	0.023[0,007)	0.023 (0.006)	0.03(0.004)	.965	.279	.041*
Soleus MV	140.27(29.35)	130.63(51.59)	167.03(56.54)	.625	.370	.018*
Soleus MV: mL/ (kg* cm)	0.034(0,01)	0.032(0.006)	0.04(0.003)	.714	.563	.683
Medial posterior soleus MV	66.53(19.44)	54.03(26.71)	66.33(28.72)	.268	.990	.007*

Medial posterior soleus MV mL/ (kg* cm)	0.016(0.006)	0.013(0.003)	0.014(0.003)	.282	.578	.392
Lateral posterior soleus MV	46.89(21.21)	50.37(19.04)	68.98(20.17)	.722	.092	.135
Lateral posterior soleus MV mL/ (kg* cm)	0.01(0.006)	0.01(0.003)	0.02(0.003)	.646	.122	.077
Medial anterior soleus MV	12.79(2.91)	13.54(6.89)	14.27(7.07)	.760	.675	.130
Medial anterior soleus MV mL/ (kg* cm)	0.003(0.002)	0.003(0.001)	0.003(0.001)	.747	.867	.259
Lateral anterior soleus MV	14.07(3.80)	12.68(7.22)	17.45(8.68)	.609	.449	.005*
Lateral anterior soleus MV mL/ (kg* cm)	0.003(0.002)	0.003(0.001)	0.004(0.000)	.662	.770	.073
Triceps surae MV	238.18(52.96)	224.49 (92.88)	293.03(108.07)	.700	.336	.014*
Triceps surae MV: mL/ (kg* cm)	0.06(0.02)	0.05(0.01)	0.06(0.005)	.806	.402	.126
Fascicle lengths (FL) mm and FL/body height, in mm	**TD** *n=8*	**CP MAL** *n=8*	**CP LAL** n=4	**TD/CP MAL** p-value	**TD/CP LAL** p-value	**CP MAL/LAL** p-value
MG FL	27.61(4.70)	27.96(8.42)	28.77(7.07)	.918	.780	.124
MG FL / mm	0.019(0.004)	0.020(0.006)	0.020(0.004)	.724	.755	.235
LG FL	17.30(6.56)	21.00(14.26)	16.29(7.10)	.519	.822l	.832
LG FL /mm	0.013(0.005)	0.015(0.009)	0.011(0.005)	.473	.761	.975
Medial Posterior Soleus FL †	25.91(6.74)	29.03(16.17)	32.62 [20.78,38.91] †	.627	.461 †	.715†
Lateral Posterior Soleus FL	14.56(4.07)	21.34(6.23)	21.97(7.41)	.024*	.137	.715

Medial Anterior Soleus FL	15.19 [12.52, 19.16] †	14.70 [12.65, 32.60] †	20.18 [10.70, 21.25] †	.721†	.154†	1.000
Lateral Anterior soleus FL	15.70(5.66)	18.10(8.02)	21.65 [10.95,23.95] †	.505	.368 †	1.000
Medial posterior soleus FL / mm	0.019(0.005)	0.022(0.012)	0.023(0.007)	.508	.305	.229
Lateral Posterior Soleus FL / mm	0.011(0.003)	0.016(0.004)	0.016(0.004)	.020*	.073	.992
Medial anterior soleus FL FL/ mm	0.011(0.002)	0.013(0.005)	0.013(0.003)	.248	.300	.720
Lateral anterior soleus FL / mm	0.011(0.004)	0.013(0.006)	0.014(0.003)	.435	.274	.629
Pennation angle	**TD** *n=8*	**CP MAL** *n=8*	**CP LAL** n=4	**TD/CP MAL** p-value	**TD/CP LAL** p-value	**CP MAL/LAL** p-value
MG	21.46(3.20)	17.64(2.29)	20.56(8.32)	.017*	.839	.139
LG	25.41 (6.94)	22.03(8.69)	31.82(12.83)	.404	.630	.115
Medial Posterior Soleus	16.36(4.81)	15.88(5.00)	16.87(6.71)	.847	.899	.051
Lateral Posterior Soleus	22.08(8.35)	22.77(6.48)	23.88(7.92)	.857	.727	.024*
Medial anterior Soleus	19.87(4.20)	17.13(5.07)	16.22(5.70)	.260	.311	0.223
Lateral Anterior Soleus	16.66(4.75)	14.65(5.16)	17.80(4.22)	.430	.687	0.232

Physiological cross-sectional area, cm²	**TD** *n=8*	**CP MAL** *n=8*	**CP LAL** n=4	**TD/CP MAL** p-value	**TD/CP LAL** p-value	**CP MAL/LAL** p-value
MG	28.27 (8.76)	24.88 [12.67, 33.64] †	34.58 [17.69, 35.88] †	.295	.683†	.028*
LG	19.28(6.25)	23.88(11.12)	28.57(7.39)	.329	.081	.323
Medial posterior soleus	28.83(12.20)	29.23(27.00)	25.56(14.14)	.970	.709	.072
Lateral posterior soleus	35.29(11.87)	25.87(7.63)	32.87(2.93)	.083	.601	.436
Medial anterior soleus	9.06(2.12)	8.66(3.27)	8.40(2.99)	.776	.655	.818
Lateral anterior soleus	10.04(3.56)	7.90(2.46)	8.60 [7.27,13.06]	.187	1.00†	.273
